# Prognostic Significance and Factors Associated With Worsening LV Dilatation in Heart Failure

**DOI:** 10.1002/clc.70490

**Published:** 2026-10-07

**Authors:** Tauben Averbuch, Robert J. H. Miller, Justin Ezekowitz, Jonathan G. Howlett, Finlay A. McAlister, Erik Youngson, Nowell M. Fine

**Affiliations:** ^1^ Department of Cardiac Sciences, Division of Cardiology Libin Cardiovascular Institute, Cumming School of Medicine Calgary Canada; ^2^ Department of Medicine, Canadian VIGOUR Center University of Alberta Edmonton Canada; ^3^ Data and Research Services, Alberta Support Unit and Provincial Research Data Services Alberta Health Services Calgary Canada

## Abstract

**Background:**

Left‐ventricular end‐systolic dimension (LVESD) and end‐diastolic dimension (LVEDD) are associated with adverse outcomes in heart failure (HF). The prognostic significance of a change in LVESD or LVEDD is uncertain.

**Methods:**

This retrospective cohort study included patients experiencing ≥ 1 HF hospitalization or emergency department (ED) visit between April 2008 and March 2016 and underwent ≥ 2 echocardiograms, separated by ≥ 6 months. The primary outcome was the composite all‐cause mortality, HF hospitalization, and ED visits for HF. We performed cut‐point analysis to determine the threshold increase in LVESD or LVEDD associated with all‐cause mortality. We evaluated characteristics associated with an increase in LVESD and LVEDD above threshold using multivariable logistic regression.

**Results:**

We included 8790 patients, 56.1% were male, with a mean (SD) age of 70.9 (14.5). The thresholds for change in LVESD and LVEDD associated with all‐cause mortality were −3.13% and 1.33%, respectively. Patients with ischemic cardiomyopathy and lower LVEF were at increased risk of a change in LV size above the threshold. There was a significant association between an increase in LVESD or LVEDD above threshold and the composite outcome (HR 1.13; 95% CI 1.06, 1.20; HR 1.07; 95% CI 1.01, 1.13, respectively). An increase in LVESD or LVEDD above threshold was associated with increased risk of HF hospitalization (HR 1.27; 95% CI 1.15, 1.39; HR 1.24; 95% CI 1.12, 1.36) and an increased risk of ED visits for HF (HR 1.25; 95% CI 1.15, 1.37; HR 1.16; 95% CI 1.06, 1.26).

**Conclusions:**

Small adverse changes in linear dimensions of LVESD and LVEDD are clinically significant in patients with HF.

## Graphical abstract

Among patients with a hospitalization or ED visit for HF and at least two echocardiograms at least 6 months apart, failure to decrease LVESD by at least 3.13% or an increase in LVEDD of 1.33% were associated with the composite of all‐cause death, HF hospitalization, and ED visits for HF. Figure created using Canva and Servier Medical Art. ED, emergency department; HF, heart failure; LVEDD, left ventricular end‐diastolic dimension; LVESD, left ventricular end‐systolic dimension.

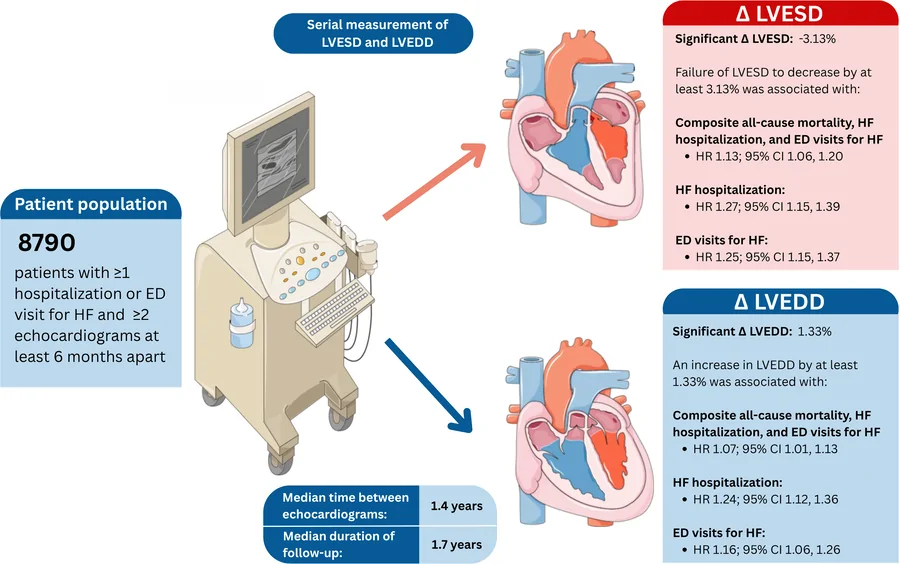

## 1 Introduction

Left ventricular dilatation is an important prognostic variable in chronic heart failure (HF). An elevated left ventricular end‐systolic dimension (LVESD) or left ventricular end‐diastolic dimension (LVEDD) has previously been shown to be associated with risk of all‐cause mortality, ventricular arrhythmia, sudden death, and deterioration of left ventricular ejection fraction (LVEF) among patients with HF with reduced ejection fraction (HFrEF) [1, 2, 3, 4]. LVESD and LVEDD have also been shown to be associated with recovery of LVEF to > 50% among patients with chronic HFrEF; in one cohort study, a decrease in LVESD to < 40 mm or LVEDD to ≤ 53 mm were associated with recovery of LVEF to > 50% on follow‐up echocardiography. LVESD and LVEDD have also been shown to predict response to coronary revascularization and cardiac resynchronization therapy (CRT) [5, 6]. However, the relationship between changes in LVESD or LVEDD and clinical outcomes is less well‐studied, and a clinically relevant change in LVESD or LVEDD has not been defined.

In a cohort of patients recently hospitalized for HF, among patients whose LVEF normalized at 6 month follow‐up, the mean LVESD decreased by 19.7%, while the mean LVEDD decreased by 6.3% [7]. Changes in LVEDD have also been associated with the ventilatory response to exercise and peak VO_2__._ A decrease in LVESD or LVEDD is associated with an improvement in New York Heart Association functional class; in one cohort study, patients with improvement in NYHA class had a mean decrease in LVEDD of 3.1 (0.7) mm and a mean (SD) decrease in LVESD of 6.6 (0.8) mm at 12−18 months of follow‐up [4]. However, the prognostic significance of minor changes in LVESD or LVEDD, smaller than what would be observed with symptomatic improvement or recovery of LVEF, have not been defined.

A change in LVESD or LVEDD of ≥ 10% has previously been used to define left ventricular reverse remodeling in the context of CRT; patients who achieve a significant reduction in internal dimensions are considered to be CRT‐responders [8, 9]. However, clinical outcomes associated with left ventricular adverse remodeling, as defined by increasing linear internal dimensions, have not been described. Furthermore, the prognostic relevance of changes in LVESD or LVEDD outside the context of device therapy, and across the spectrum of LVEF is unclear. In this study, we aimed to establish an optimal cut‐off value for an increase in LVESD or LVEDD that is associated with risk of all‐cause mortality, and identify clinical factors associated with an increase in LVESD and LVEDD. Lastly, we aimed to compare clinical outcomes between patients who experienced an increase in LVESD or LVEDD and those who did not.

## 2 Methods

### 2.1 Setting and Data Sources

We performed a retrospective cohort study of all patients living in the province of Alberta (Canada) older than 18 with a diagnosis of HF; defined as at least one HF hospitalization or ED visit with an ICD‐10 code for HF (I50.x), between April 1, 2008 and March 31, 2016 who underwent at least two echocardiograms, separated by a minimum of 6 months. The echocardiograms were performed and interpreted as part of clinical care. Only the first two echocardiograms separated by 6 months within the study period were included in the analysis. We included patients across the spectrum of LVEF. We identified baseline covariates using the ICD‐10 codes and case definitions validated in Alberta administrative databases for all hospitalizations in the 12 months preceding the index echocardiogram.

We linked our study sample to the echocardiography database of the two main HF referral centers in the province, the Mazankowski Heart Institute (Edmonton, Alberta) and the Libin Cardiovascular Institute (Calgary, Alberta) using each patient's personal health identifier. We included a mix of inpatient and outpatient echocardiograms. We removed patients who did not have at least two echocardiograms performed at least 6 months apart. We also excluded patients who did not have LVESD or LVEDD recorded. In order to comply with Alberta's Health Information Protection Act, the data set for this study is not publicly available.

### 2.2 Outcomes

Our primary outcome was the composite of all‐cause mortality, HF hospitalization, and emergency department (ED) presentations for HF. Our secondary outcomes included the individual components of the composite outcome.

### 2.3 Statistical Analysis

Patient characteristics were summarized with continuous variables expressed as means with standard deviations where normally distributed, and medians with interquartile ranges (IQR) where not using the Shapiro−Wilk test. Categorical variables were expressed as frequencies and percentages. Categorical variables were compared with the Chi‐square or Fisher's exact test, and continuous variables with the Student's *t*‐test or Wilcoxon rank sums, as appropriate. To determine the optimal cut‐off value for an increase in LVESD and LVEDD, we performed receiver‐operating characteristic analysis for all‐cause mortality based on the Youden index, consistent with a previous analysis we performed with the same data set [10]. We evaluated characteristics associated with an increase in LVESD and LVEDD using two separate multivariable logistic regression models. The regression models contained clinically relevant variables as well as all variables with statistically significant differences between those with progressive LV dilatation and those without as candidate variables. Backwards stepwise variable selection was used to develop the final parsimonious multivariable model.

Clinical outcomes were compared among those who experienced an increase in LV dimensions above the threshold value and those who did not using Cox‐proportional hazards models and Kaplan−Meier curves. Outcomes that included death were modeled with Cox‐proportional hazards models, otherwise, Fine−Gray sub‐distribution hazard models were developed, with death as a competing risk. Time to first event was assessed for all outcomes, with follow‐up starting from the date of the second echocardiogram. Follow‐up was censored at March 31, 2017 or date of known migration out of the province. For each outcome, we calculated unadjusted hazard ratios (HR), age and sex adjusted HR, and HR adjusted for age, sex, site, time between the two echocardiograms, and statistically significant variables obtained using a backward stepwise selection. The variables included in the final parsimonious model for mortality were used to evaluate all other outcomes. The fully adjusted models included the time between echocardiograms, baseline linear dimensions, ejection fraction, clinical comorbidities, device, and pharmacologic HF therapies. Variables measured at baseline refer to their values/presence at the time of the first echocardiogram. Variables measured at follow‐up refer to their values/presence at the time of the second echocardiogram. Comorbidities were determined at the time of the second echocardiogram using administrative databases and ICD‐10‐CA codes. We performed a sensitivity analysis where we used restrictive cubic splines for the unadjusted analysis for the association between the absolute change in LVESD or LVEDD (continuous) and the primary composite outcome. Lastly, we performed a separate sensitivity analysis to assess the adjusted association between a 3 mm increase in LVESD or a 3 mm increase in LVEDD and each of the clinical outcomes, to reflect changes in linear dimensions greater than the expected measurement error. The analysis of 3 mm changes in linear dimensions was adjusted for the same variables as the primary analysis (Central Illustration iillustration 1, 1).

**Central iillustration 1:**
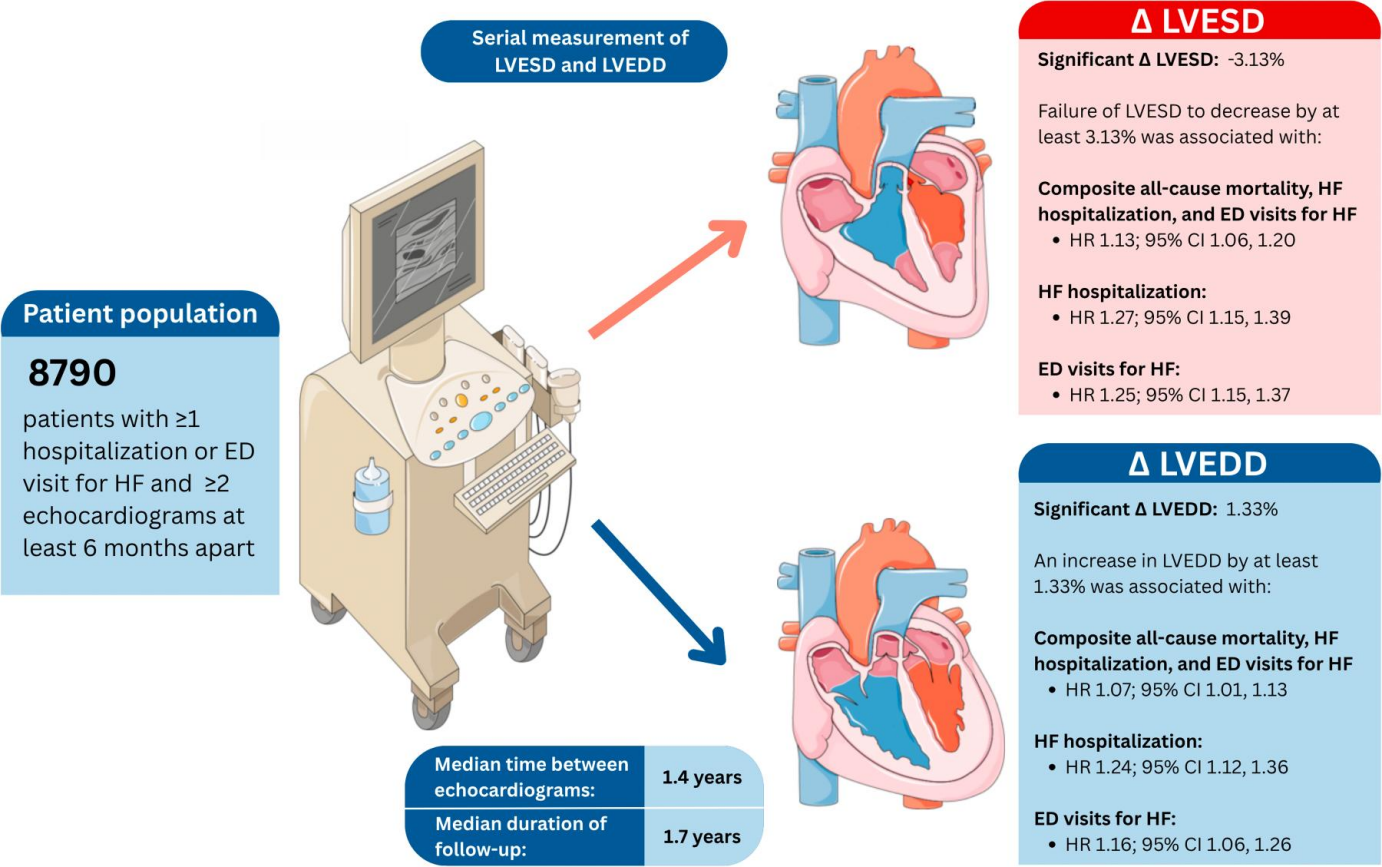
The association between changes in LVESD or LVEDD and clinical outcomes. Among patients with a hospitalization or ED visit for HF and at least two echocardiograms at least 6 months apart, failure to achieve a ≥ 3.13% decrease in LVESD or an increase in LVEDD of ≥ 1.33% were associated with the composite of all‐cause death, HF hospitalization, and ED visits for HF. Figure created using Canva and Servier Medical Art. ED, emergency department; HF, heart failure; LVEDD, left ventricular end‐diastolic dimension; LVESD, left ventricular end‐systolic dimension.

All statistical tests were two‐sided with a *p* value less than 0.05 considered significant. Statistical analyses were performed using StataSE 17 (Statacorp, College Station, TX). This study complies with the Declaration of Helsinki and was approved by the conjoint health research ethics board at the University of Calgary and the University of Alberta with a waiver for consent (Approval code: REB16‐1754_MOD1; Approval date: September 11, 2025).

## 3 Results

### 3.1 Patient Demographics

There were 8790 patients included in this study. The mean (SD) age at follow‐up was 70.9 (14.5), and 56.1% of patients were male (Table 1). The burden of comorbidities was high, with 36.2% having diabetes mellitus, 41.7% having chronic kidney disease, and 44.1% ischemic heart disease. Background uptake of guideline‐directed medical therapy was high (Table 1).

**Table 1:** Baseline and clinical characteristics, separated by increase in LVESD or LVEDD.

	No increase in LVESD (*N* = 3808)	Increase in LVESDa (*N* = 4982)	*p* value	No increase in LVEDD (*N* = 4820)	Increase in LVEDDb (*N* = 3970)	*p* value
Age at follow‐up in years, mean (SD)	70.2 (14.8)	71.41 (14.3)	< 0.01	71.0 (14.5)	70.74 (14.6)	0.43
Male	2128 (55.9%)	2802 (56.2%)	0.74	2700 (56.0%)	2230 (56.2%)	0.88
Income quintile			0.04			0.09
1 (highest)	724 (19.0%)	982 (19.7%)		956 (19.8%)	750 (18.9%)	
2	726 (19.1%)	983 (19.7%)		930 (19.3%)	779 (19.6%)	
3	783 (20.6%)	930 (18.7%)		953 (19.8%)	760 (19.1%)	
4	762 (20.0%)	963 (19.3%)		966 (20.0%)	759 (%)	
5 (lowest)	704 (18.5%)	1009 (20.3%)		887 (18.4%)	826 (%)	
Missing	109 (2.9%)	115 (2.3%)		128 (2.6%)	96 (%)	
Comorbidities						
Atrial fibrillation, *n* (%)	1579 (41.47%)	2089 (41.93%)	0.66	2030 (42.1%)	1638 (41.3%)	0.42
Ischemic heart disease, *n* (%)	1613 (42.36%)	2260 (45.36%)	0.01	2066 (42.9%)	1807 (45.5%)	0.01
Chronic kidney disease, *n* (%)	1532 (40.2%)	2136 (42.9%)	0.01	2012 (41.7%)	1656 (41.7%)	0.98
Chronic obstructive pulmonary disease, *n* (%)	1062 (27.9%)	1407 (28.2%)	0.72	1358 (28.2%)	1111 (28.0%)	0.84
Dementia, *n* (%)	219 (5.8%)	298 (6.0%)	0.65	291 (6.0%)	226 (5.7%)	0.49
Cancer, *n* (%)	328 (8.6%)	459 (9.2%)	0.33	413 (8.6%)	374 (9.4%)	0.16
Anemia, *n* (%)	386 (10.1%)	536 (10.8%)	0.35	500 (10.4%)	422 (10.6%)	0.70
Diabetes, *n* (%)	1325 (34.8%)	1857 (37.3%)	0.02	1711 (35.5%)	1471 (37.1%)	0.13
Hypertension, *n* (%)	2424 (63.7%)	3414 (68.5%)	< 0.01	3146 (65.3%)	2692 (67.8%)	0.01
Depression, *n* (%)	332 (8.7%)	433 (8.7%)	0.96	414 (8.6%)	351 (8.8%)	0.68
Previous stroke or transient ischemic attack, *n* (%)	339 (8.9%)	448 (9.0%)	0.88	429 (8.9%)	358 (9.0%)	0.85
Prescribed medications						
Prescribed ARB, *n* (%)	1032 (27.1%)	1433 (28.8%)	0.09	1343 (27.9%)	1122 (28.3%)	0.68
Prescribed ACEi, *n* (%)	2253 (59.2%)	2738 (55.0%)	< 0.01	2800 (58.1%)	2191 (55.2%)	0.01
Prescribed beta‐blocker, *n* (%)	2946 (77.4%)	3630 (72.9%)	< 0.01	3704 (76.9%)	2872 (72.3%)	< 0.01
Prescribed ARNI, *n* (%)	0 (0.0%)	0 (0.0%)		0 (0.00%)	0 (0.0%)	
Prescribed MRA, *n* (%)	1019 (26.8%)	1090 (21.9%)	< 0.01	1196 (24.8%)	913 (23.0%)	0.05
Prescribed ARB at follow‐up, *n* (%)	945 (24.8%)	1305 (26.2%)	0.14	1226 (25.4%)	1024 (25.8%)	0.70
Prescribed ACEi at follow‐up, *n* (%)	2071 (54.4%)	2506 (50.3%)	< 0.01	2563 (53.2%)	2014 (50.7%)	0.02
Prescribed beta‐blocker at follow‐up, *n* (%)	2796 (73.4%)	3424 (68.7%)	< 0.01	3517 (73.0%)	2703 (68.1%)	< 0.01
Prescribed ARNI at follow‐up, *n* (%)	0 (0.0%)	0 (0.0%)		0 (0.0%)	0 (0.0%)	
Prescribed MRA at follow‐up, *n* (%)	877 (23.0%)	913 (18.3%)	< 0.01	1,017 (21.1%)	773 (19.5%)	0.06
Echocardiographic parameters						
Index LVEF, mean (SD)	44.8 (15.0)	48.8 (13.1)	< 0.01	45.9 (14.7)	48.5 (13.3)	< 0.01
Follow‐up LVEF, mean (SD)	49.5 (12.3)	45.6 (14.3)	< 0.01	48.2 (12.9)	46.2 (14.3)	< 0.01
Index LVESD, mean (SD)	3.9 (1.2)	3.3 (1.1)	< 0.01	3.7 (1.2)	3.3 (1.1)	< 0.01
Index LVEDD, mean (SD)	5.2 (1.0)	4.9 (0.9)	< 0.01	5.2 (0.9)	4.7 (0.9)	< 0.01
Follow‐up LVESD, mean (SD)	3.1 (1.0)	3.8 (1.1)	< 0.01	3.4 (1.1)	3.8 (1.2)	< 0.01
Follow‐up LVEDD, mean (SD)	4.8 (0.9)	5.1 (0.9)	< 0.01	4.7 (0.9)	5.2 (0.9)	< 0.01
Index MR severity, *n* (%)			< 0.01			< 0.01
Mild, mild to moderate	1599 (42.0%)	1895 (38.0%)		2018 (41.9)	1476 (37.2)	
Moderate, moderate to severe	484 (12.7%)	478 (9.6%)		596 (12.4)	366 (9.2)	
Severe	76 (2.0%)	88 (1.8%)		115 (2.4)	49 (1.2)	
Clinical outcomes						
Composite all‐cause mortality, Hospitalization for HF, and ED visits for HF, *n* (%)	2144 (56.3%)	3004 (60.3%)	< 0.01	2765 (57.4%)	2383 (60.0%)	0.01
All‐cause mortality, *n* (%)	1637 (43.0%)	2260 (45.4%)	0.04	2096 (43.5%)	1801 (45.4%)	0.08
Hospitalization for HF, *n* (%)	812 (21.3%)	1245 (25.0%)	< 0.01	1069 (22.2%)	988 (24.9%)	< 0.01
ED visit for HF, %	944 (24.8%)	1417 (28.4%)	< 0.01	1266 (26.3%)	1095 (27.6%)	0.17

Abbreviations: ACE, angiotensin‐converting enzyme; ARB, angiotensin receptor blocker; ARNI, angiotensin receptor neprilysin inhibitor; ED, emergency department; HF, heart failure; LVEDD, left ventricular end‐diastolic dimension; LVEF, left ventricular ejection fraction; LVESD, left ventricular end‐systolic dimension; MR, mitral regurgitation; MRA, mineralocorticoid receptor antagonist; SD, standard deviation.

^a^ An increase in LVESD was defined as an increase ≥ −3.13% between echocardiograms ≥ 6 months apart

^b^ An increase in LVEDD was defined as a ≥ 1.33% increase in LVEDD between echocardiograms ≥ 6 months apart.

### 3.2 Echocardiographic Parameters

The median time between echocardiograms was 1.40 (IQR: 0.89, 2.42) years. The mean (SD) LVEF was 47.1 (14.1) % at the time of the initial echocardiogram, and 47.3 (13.6) % at the time of the follow‐up echocardiogram. The mean (SD) LVESD on the index echocardiogram was 3.52 (1.17) and 3.53 (1.14) cm on the follow‐up echocardiogram (Table 1). The mean (SD) LVEDD on the index echocardiogram was 4.98 (0.94) and 4.96 (0.93) cm on the follow‐up echocardiogram (Supporting Information S1: Table S1). The mean (SD) right ventricular systolic pressure (RVSP) at the index echocardiogram was 43.95 (16.12) mmHg. The mean (SD) RVSP at the follow‐up echocardiogram was 44.88 (16.85) mmHg.

### 3.3 Clinical Outcomes

The median (IQR) duration of follow‐up was 1.7 (0.6, 3.4) years. There were 5148 (58.6%) all‐cause deaths, HF readmissions, or ED visits for HF over the course of follow‐up. The individual component outcomes were common; there were 3897 (44.3%) all‐cause deaths, 2057 (23.4%) HF hospitalizations, and 2361 (26.9%) ED visits for HF (Table 1).

### 3.4 Cut‐Point Analysis and Variables Associated With an Increase in LVESD and LVEDD

Analysis of the percentage change in LVESD for all‐cause mortality revealed a threshold of −3.13% such that patients who failed to achieve a ≥ 3.13% decrease in LVESD were at increased risk of all‐cause mortality. A 3.13% relative change in the mean baseline LVESD represents an absolute change of 1.10 mm. The cut‐point for the percentage change in LVEDD for all‐cause mortality was 1.33%, such that patients whose LVEDD increased by more than 1.33% were at increased risk of all‐cause mortality. A 1.33% relative change in the mean baseline LVEDD represents an absolute change of 0.66 mm. In a multivariable logistic regression model, variables significantly associated with failure to achieve a ≥ 3.13% decrease in LVESD included the index severity of mitral regurgitation, index LVESD, index LVEF, and history of ischemic heart disease (Table 2); the same variables were significantly associated with a ≥ 1.33% increase in LVEDD (Table 3).

**Table 2:** Multivariable regression model of variables associated with a failure to achieve a ≥ 3.13% decrease in LVESD.

Variable	Odds ratio (95% CI)
Index mitral regurgitation severity	
Mild, mild to moderate	0.87 (0.79, 0.96)
Moderate, moderate to severe	0.89 (0.77, 1.04)
Severe	1.25 (0.89, 1.75)
Age	1.00 (0.99, 1.00)
Index LVESD	0.28 (0.25, 0.31)
Index LVEDD	2.11 (1.91, 2.34)
Ischemic heart disease	1.28 (1.16, 1.40)
Prescribed a mineralocorticoid receptor antagonist at baseline	1.10 (0.99, 1.24)
Index LVEF	0.97 (0.97, 0.98)

Abbreviations: LVEDD, left ventricular end diastolic dimension; LVEF, left ventricular ejection fraction; LVESD, left ventricular end systolic dimension.

**Table 3:** Multivariable regression model of variables associated with a ≥ 1.33% increase in LVEDD.

Variable	Odds ratio (95% CI)
Index mitral regurgitation severity	
Mild, mild to moderate	0.82 (0.74, 0.91)
Moderate, moderate to severe	0.82 (0.70, 0.97)
Severe	0.85 (0.59, 1.22)
Ischemic heart disease	1.29 (1.17, 1.42)
Index LVESD	1.59 (1.44, 1.75)
Age	0.99 (0.98, 0.99)
Index LVEDD	0.25 (0.23, 0.28)
Prescribed a beta‐blocker at baseline	0.93 (0.83, 1.03)
Prescribed a mineralocorticoid receptor antagonist at baseline	1.22 (1.09, 1.37)
LVEF	0.99 (0.99, 1.00)

Abbreviations: LVEDD, left ventricular end diastolic dimension; LVEF, left ventricular ejection fraction; LVESD, left ventricular end systolic dimension.

### 3.5 Association Between an Increase in LVESD and Clinical Outcomes

There were 4982 (56.7%) patients who failed to achieve at a ≥ 3.13% decrease in LVESD between echocardiograms. In the unadjusted (HR 1.15, 95% CI 1.08, 1.21) and fully adjusted model (HR 1.13, 95% CI 1.06, 1.20), a less than 3.13% decrease in LVESD was significantly associated with the time to the composite outcome (Central Illustration iillustration 1, 1, Figure iillustration 1, 1, Table 4, Figure S1A‐B).

**Figure 1:**
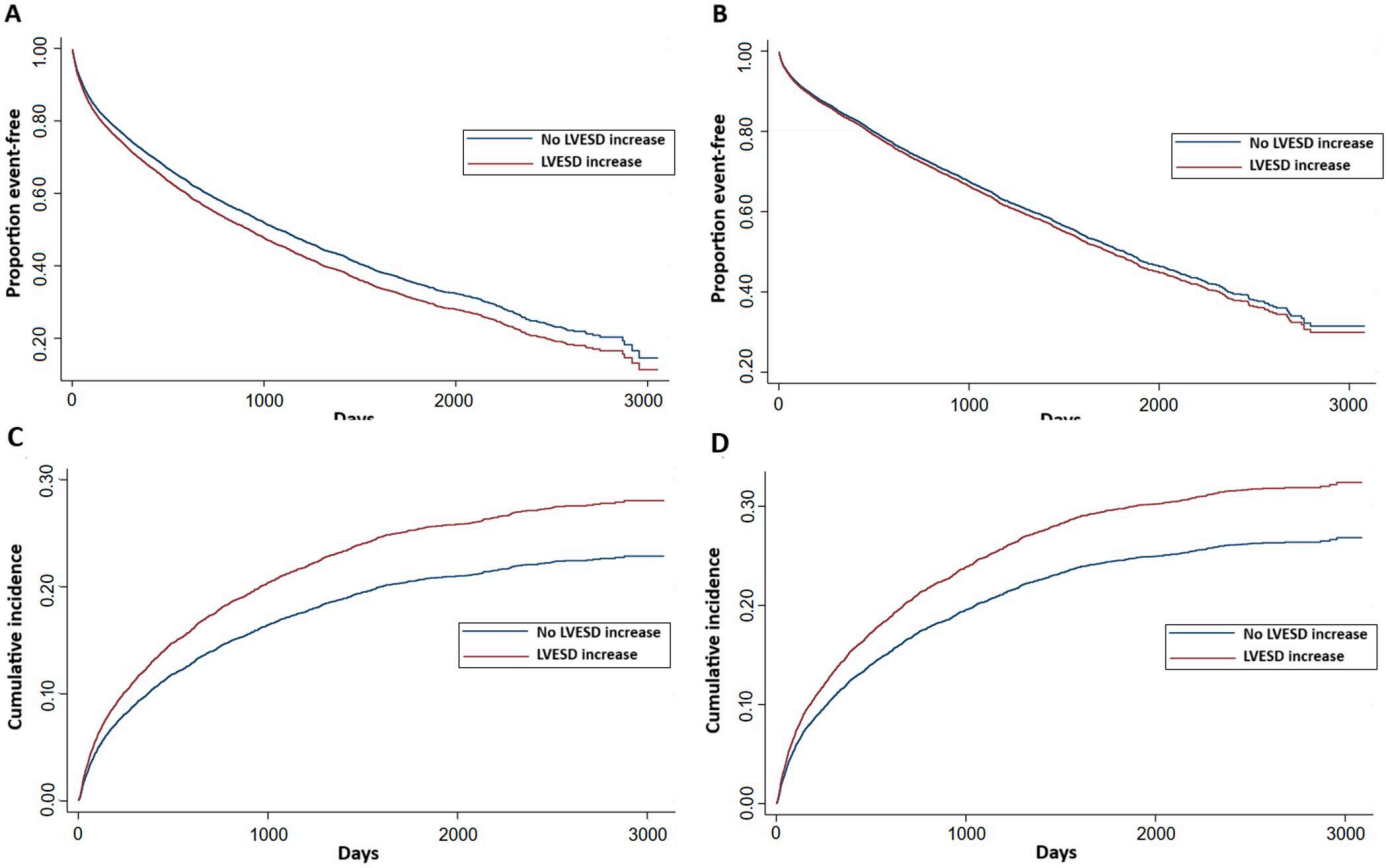
The associations between a failure to achieve a ≥ 3.13% decrease in LVESD and clinical outcomes, fully adjusted models. (A) Composite all‐cause death, HF readmission, or ED visits for HF; (B) all‐cause death; (C) HF readmission; and (D) ED visits for HF. Patients with an increase in LVESD above threshold experienced greater composite all‐cause death, HF hospitalization, or ED visits for HF, as well as the component outcomes, but not all‐cause death alone. ED, emergency department; HF, heart failure; LVESD, left ventricular end‐systolic dimension.

**Table 4:** Association between failure to achieve a ≥ 3.13% decrease in LVESD and composite all‐cause mortality, HF readmission, and ED visits for HF.

Variable	Hazard ratio (95% CI)
LVESD	1.13 (1.06, 1.20)
Index LVEF	1.00 (1.00, 1.00)
Index LVESD	1.05 (1.01, 1.10)
Age	1.03 (1.03, 1.03)
Male sex	0.98 (0.93, 1.04)
Ischemic heart disease	1.12 (1.05, 1.19)
Atrial fibrillation	1.23 (1.16, 1.30)
Index mitral regurgitation severity	
Mild, mild to moderate	1.08 (1.02, 1.15)
Moderate, moderate to severe	1.25 (1.13, 1.37)
Severe	1.04 (0.84, 1.28)
Chronic obstructive pulmonary disease	1.40 (1.31, 1.48)
Chronic kidney disease	1.57 (1.48, 1.67)
Hypertension	1.02 (0.95, 1.09)
Dementia	1.32 (1.19, 1.47)
Cancer, any	1.32 (1.21, 1.44)
Anemia	1.19 (1.09, 1.29)
Diabetes	1.36 (1.28, 1.45)
Depression	1.15 (1.05, 1.26)
CRT	1.01 (0.64, 1.62)
ICD	0.81 (0.68, 0.97)
Permanent pacemaker	0.95 (0.83, 1.08)
Prescribed any ACE inhibitor or ARB at follow‐up	0.91 (0.85, 0.97)
Prescribed any beta‐blocker at follow‐up	0.87 (0.82, 0.93)

*Note:* An increase in LVESD was defined as an increase ≥ −3.13% between echocardiograms ≥ 6 months apart

Abbreviations: ACE, angiotensin converting enzyme; CRT, cardiac resynchronization therapy; ICD, implantable cardioverter‐defibrillator; LVEF, left ventricular ejection fraction; LVESD, Left ventricular end systolic dimension.

In the unadjusted (HR 1.09; 95% CI 1.02, 1.16) but not fully adjusted analyses (HR 1.04, 95% CI 0.98, 1.12), there was a significant association between a decrease of less than 3.13% in LVESD and time to all‐cause death (Figure iillustration 1, 1, Figure S1C‐D, Supporting Information S1: Table S1).

A decrease of less than 3.13% in LVESD was significantly associated with time to first HF hospitalization in the unadjusted (HR 1.21; 95% CI 1.10, 1.32) and fully adjusted models (HR 1.27, 95% CI 1.15, 1.39) (Figure iillustration 1, 1, Figure S2A‐B, Supporting Information S1: Table S1). A decrease of less than 3.13% in LVESD was also significantly associated with time to first ED visit for HF in the unadjusted (HR 1.19; 95% CI 1.09, 1.29) and fully‐adjusted models (HR 1.25, 95% CI 1.15, 1.37, respectively) (Figure iillustration 1, 1, Figure S2C‐D, Supporting Information S1: Table S1).

### 3.6 Association Between an Increase in LVEDD and Clinical Outcomes

There were 3970 (45.2%) patients with a ≥ 1.33% increase in LVEDD between echocardiograms. A ≥ 1.33% increase in LVEDD was significantly associated with the time to the composite outcome in the unadjusted (HR 1.07, 95% CI 1.01, 1.13) and fully adjusted models (HR 1.07, 95% CI 1.01, 1.13) (Figure 2A, Figure S3A‐B, Table 5).

**Figure 2:**
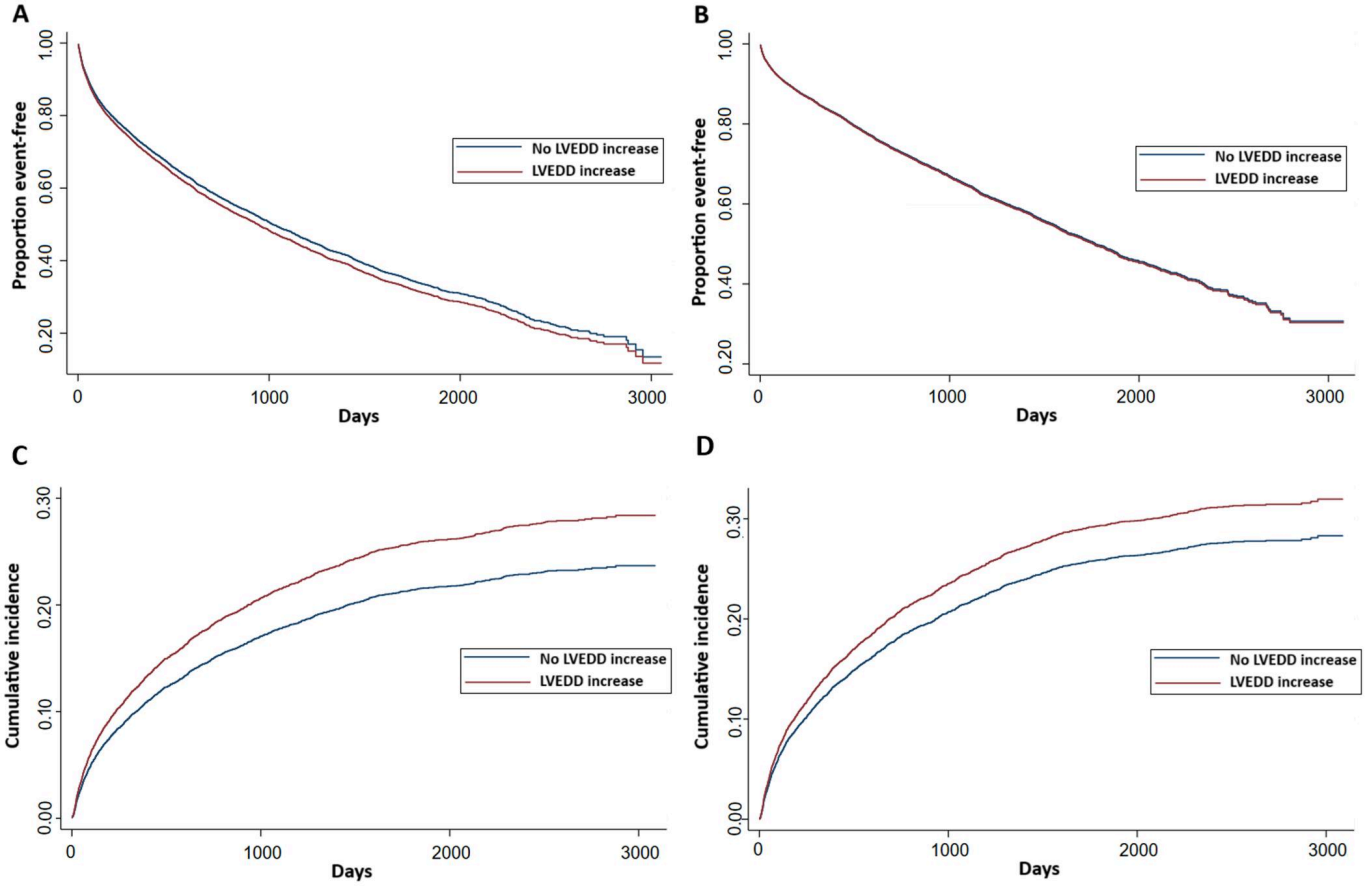
The association between an increase in LVEDD and clinical outcomes, fully adjusted models. (A) Composite all‐cause death, HF readmission, or ED visits for HF; (B) all‐cause death; (C) HF readmission; (D) ED visits for HF. Patients with an increase in LVEDD were at greater risk of composite all‐cause death, HF readmission, or ED visits for HF, as well as the component outcomes, but not all‐cause death alone. An increase in LVEDD was defined as a ≥ 1.33% increase in LVEDD between echocardiograms ≥ 6 months apart. ED, emergency department; HF, heart failure; LVEDD, left ventricular end‐diastolic dimension.

**Table 5:** Association between LVEDD and composite all‐cause mortality, HF readmission, and ED visits for HF.

Variable	Hazard ratio (95% CI)
LVEDD increase	1.07 (1.01, 1.13)
Index LVEF	1.00 (1.00, 1.00)
Index LVEDD	1.01 (0.97, 1.06)
Age	1.03 (1.03, 1.03)
Male sex	1.00 (0.94, 1.06)
Ischemic heart disease	1.12 (1.05, 1.19)
Atrial fibrillation	1.23 (1.16, 1.30)
Index mitral regurgitation severity	
Mild, mild to moderate	1.09 (1.02, 1.16)
Moderate, moderate to severe	1.26 (1.15, 1.38)
Severe	1.07 (0.86, 1.33)
Chronic obstructive pulmonary disease	1.39 (1.31, 1.48)
Hypertension	1.02 (0.95, 1.09)
Chronic kidney disease	1.57 (1.48, 1.67)
Dementia	1.32 (1.19, 1.46)
Cancer (any)	1.31 (1.20, 1.43)
Anemia	1.19 (1.09, 1.29)
Diabetes	1.36 (1.28, 1.45)
Depression	1.14 (1.04, 1.26)
CRT	1.04 (0.65, 1.67)
ICD	0.84 (0.70, 1.00)
Permanent pacemaker	0.95 (0.84, 1.08)
Prescribed any ACE inhibitor or ARB at follow‐up	0.90 (0.85, 0.97)
Prescribed any beta‐blocker at follow‐up	0.87 (0.82, 0.93)

*Note:* An increase in LVEDD was defined as a ≥ 1.33% increase in LVEDD between echocardiograms ≥ 6 months apart.

Abbreviations: ACE, angiotensin converting enzyme; ARB, angiotensin receptor blocker; CRT, cardiac resynchronization therapy; ICD, implantable cardioverter‐defibrillator; LVEDD, left ventricular end diastolic dimension; LVEF, left ventricular ejection fraction.

There was no significant association between a ≥ 1.33% increase in LVEDD and time to all‐cause death in the unadjusted (HR 1.05; 95% CI 0.98, 1.12) and fully adjusted models (HR 1.01, 95% CI 0.94, 1.08) (Figure 2B, Figure S3C‐D, Supporting Information S1: Table S2). There was a significant association between a ≥ 1.33% increase in LVEDD and time to HF readmission in the unadjusted (HR 1.14, 95% CI 1.04, 1.24) and fully‐adjusted models (HR 1.24; 95% CI 1.12, 1.36) (Figure 2C, Figure S4A‐B, Supporting Information S1: Table S2).

There was a significant association between a ≥ 1.33% increase in LVEDD and time to first ED visit for HF in the fully‐adjusted (HR 1.16, 95% CI 1.06, 1.26) but not unadjusted (HR 1.05, 95% CI 0.97, 1.14) model (Figure 2D, Figure S4C‐D, Supporting Information S1: Table S2).

### 3.7 Sensitivity Analysis

Similar to the primary analysis, the restricted cubic splines analysis found that small changes in LVESD and LVEDD were prognostically relevant. In the unadjusted restricted cubic splines analysis, an absolute decrease in LVESD was associated with decreasing risk, with no significant increase in patients with increasing LVESD (Figure 3). Similarly, a decrease in LVEDD was associated with decreasing risk, with no significant increase in patients with increasing LVEDD (Figure 4). Similar results were seen in the multivariable model (Figures S5‐6).

**Figure 3:**
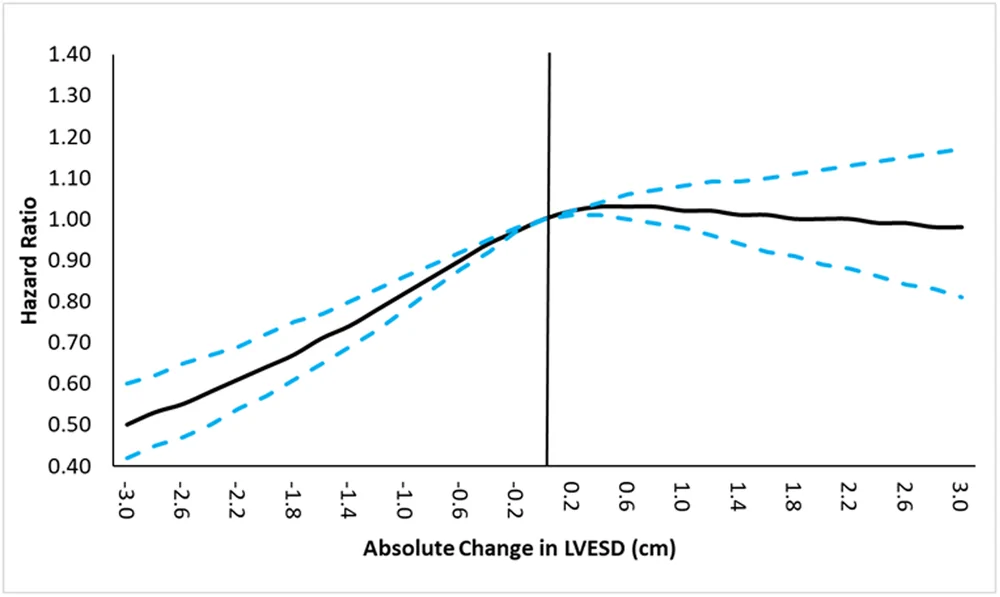
The association between the absolute change in LVESD and the composite of all‐cause death, HF hospitalization, or ED visits for HF. The analysis was performed using restricted cubic splines. There was a significant decrease in the risk of the composite outcome in patients with decreasing LVEDD. ED, emergency department; HF, heart failure; LVESD, left ventricular end systolic dimension.

**Figure 4:**
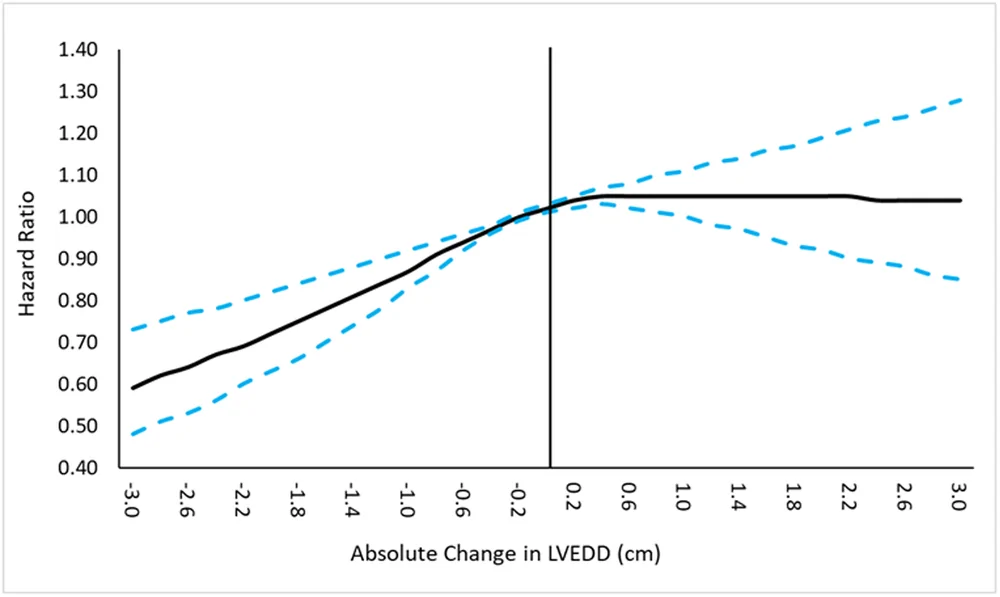
The association between the absolute change in LVEDD and the composite of all‐cause death, HF hospitalization, or ED visits for HF. The analysis was performed using restricted cubic splines. There was a significant decrease in the risk of the composite outcome in patients with decreasing LVEDD. ED, emergency department; HF, heart failure; LVEDD, left ventricular end diastolic dimension.

In the fully adjusted models, there was a significant association between a 3 mm increase in LVESD and the primary composite outcome (HR 1.09; 95% CI 1.02, 1.15), HF hospitalization (HR 1.22; 95% CI 1.11, 1.34), and ED visits for HF (HR 1.15; 95% CI 1.05, 1.26), but not all‐cause mortality (HR 0.99; 95% CI 0.93, 1.06) (Supporting Information S1: Table S3). Similarly, there was a significant association between a 3 mm increase in LVEDD and HF hospitalization (HR 1.21; 95% CI 1.10, 1.34) and ED visits for HF (HR 1.19; 95% CI 1.08, 1.30) but not all‐cause mortality (HR 0.97; 95% CI 0.90, 1.04), with a trend toward significance for the primary composite outcome (HR 1.06; 95% CI 1.00, 1.13) (Supporting Information S1: Table S4).

## 4 Discussion

In this retrospective cohort study of patients hospitalized for HF, we found that a prognostically relevant change in LVESD between echocardiograms performed ≥ 6 months apart was −3.13%, while a prognostically relevant change in LVEDD was ≥ 1.33%. Both an increase in LVEDD and a failure to achieve a ≥ 3.13% reduction in LVESD were significantly associated with the composite of all‐cause death, HF hospitalization, and ED visits, as well as their component outcomes, with the exception of mortality.

We found that the variables most strongly associated with a prognostically relevant change in LVESD were the index LVESD, index LVEDD, and history of ischemic heart disease, while the variables most strongly associated with a prognostically relevant change in LVEDD were largely similar, with the addition of prescription of a beta‐blocker or mineralocorticoid receptor antagonist at baseline. In a prior analysis of the same cohort that investigated differences between patients with and without a ≥ 10% increase in LVEF, variables associated with LVEF recovery included age, female sex, hypertension, use of hydralazine, and baseline ejection fraction [10]. Baseline characteristics associated with left ventricular adverse remodeling rather than reverse remodeling may differ.

We found that a smaller change in LVESD or LVEDD was prognostically relevant than the predefined changes used in the literature. There is a dearth of evidence regarding the prognostic relevance of serial changes in LVESD or LVEDD in chronic HF outside of the context of interventions such as CRT or coronary revascularization [11, 12]. Other studies on the serial measurement of LVESD and LVEDD have focused on the relationship between LVESD or LVEDD and the recovery of LVEF. In one retrospective study of patients with new systolic dysfunction who underwent serial echocardiograms, patients who did not have reverse remodeling (improvement in LVEF to > 35%) had a mean change in indexed LVESD of 1.7% and indexed LVEDD of 0.7% between echocardiograms, whereas patients with reverse remodeling had a mean change in indexed LVESD of −15.1% and in indexed LVEDD of −6.3%. In contrast, a more modest change in LVESD and LVEDD was prognostically relevant in our cohort; a greater percentage change in LVESD or LVEDD would be expected in the context of LVEF recovery, given their correlation with LVEF.

Our study adds to the literature regarding risk prediction in HF and specifically adds to our understanding of the relationship between the trajectory of LVESD and LVEDD and clinical outcomes following HF hospitalization. The significant association between modest changes in LVESD or LVEDD and HF hospitalization is consistent with prior literature demonstrating that patients who are rehospitalized for HF are most likely to experience an increase in LVEDD, while stable patients tend to experience a reduction in LVEDD [7]. The changes in LVESD and LVEDD that were prognostically relevant in our cohort were small (1.10 mm for LVESD and 0.66 mm for LVEDD) and within the range of measurement error for echocardiography. Our finding that patients whose linear dimensions remain stable are at increased risk of adverse events mirrors the relationship between improvements in LVEF and clinical outcomes. Patients with stable LVEF and chronic HF have considerably higher event rates than those with modest improvements in LVEF. In one cohort, death or HF hospitalization at 12 months occurred in 26.5% of patients with no improvement in LVEF, and 14.3% in patients with ≥ 5% increase in LVEF. While our cut‐point analysis found that small changes in linear dimensions were prognostically relevant, our sensitivity analysis demonstrated progressively lower risk of clinical deterioration with greater reductions in linear dimensions, concordant with the existing literature.

In our sensitivity analysis, we found that changes of 3 mm—just above the expected measurement error for linear dimensions—were significantly associated with increased risk of HF hospitalization or ED visits for HF [13]. By comparison, our primary analysis found that the risk of deterioration with stable linear dimensions is of a similar magnitude to patients whose linear dimensions increase by 3 mm—suggesting that risk may increase even before subtle changes in linear dimensions are detectable. Accordingly, patients whose LVEF or linear dimensions do not improve on serial imaging should prompt closer review as they may represent a higher risk group who have a limited window of opportunity to intensify therapy and prevent subsequent echocardiographic and clinical deterioration. Serial changes in LVESD and LVEDD could be included in risk prediction models to better identify vulnerable patients, similar to previous risk prediction models that include baseline internal dimensions [14].

## 5 Limitations

There are important limitations of our study to acknowledge. The echocardiograms were performed as part of clinical care and not by study personnel. We were not able to assess the inter‐observer reliability of measurements. Additionally, selection and ascertainment bias limit the generalizability of our findings. The timing and indications for echocardiography were not standardized; patients who experienced clinical deterioration may have been more likely to undergo serial echocardiograms. Additionally, serial imaging may have prompted further interventions, such as device, interventional, or medical therapy that could not be completely accounted for by our analysis. Left ventricular diastolic and systolic volumes were unavailable for many patients and were not adjusted for in our analysis. We did not have access to patients’ laboratory values, such as creatinine or markers of congestion, such as NT‐proBNP, recognizing the impact of volume status on left ventricular distension. We were not able to compare echocardiographic to MRI measurements. We could not account for the dosages of guideline‐directed medical therapy or for patient adherence in our analysis. Furthermore, we did not account for the context in which echocardiograms occurred, such as in routine follow‐up or clinical deterioration which could influence subsequent clinical outcomes. Outcomes were determined via ICD‐10 codes; we were not able to independently verify clinical diagnoses. Our patient population had predominantly mild systolic dysfunction with non‐remodeled left ventricles; patients with severely reduced EF or severe LV dilatation were underrepresented. Lastly, while the cut‐point approach allows for greater ease of use, dichotomization can overestimate the prognostic performance of the model. External validation would add greater confidence in the strength of the association between changes in linear dimensions and clinical outcomes, as well as the generalizability of the cut‐points established in our analysis. In the absence of external validation, serial changes in linear measurements should not guide treatment.

## 6 Conclusion

Overall, we found that a failure to achieve a ≥ 3.13% decrease in LVESD or a greater than 1.33% increase in LVEDD on echocardiograms performed ≥ 6 months apart represented the optimal cut‐point for all‐cause mortality of similar magnitude for both parameters. Patients with ischemic cardiomyopathy and decreased LVEF were the most likely to experience a progressive increase in LVESD or LVEDD. An increase in LVEDD or LVESD was significantly associated with the risk of HF hospitalization or ED visits for HF, and may provide additional prognostic information in patients with chronic HF and a prior HF hospitalization.

## Author Contributions

T.A., N.F., and R.J.H.M. conceived the study idea, performed the analysis, drafted, and edited the manuscript. E.Y. contributed to the analysis and edited the manuscript. J.E., J.G.H., and F.A.M. edited the manuscript.

## Funding

The authors have nothing to report.

## Conflicts of Interest

The authors declare no conflicts of interest.

## Supporting information


Supporting File

**Figure S1:**The association between an increase in LVESD and composite all‐cause death, HF readmission, or ED visits for HF, and an increase in LVESD and death, unadjusted and adjusted for age and sex

**Figure S2:** The association between an increase in LVESD and HF hospitalization, and an increase in LVESD and ED visits for HF, unadjusted and age and sex‐adjusted models

**Figure S3:** The association between an increase in LVEDD and composite all‐cause death, HF readmission, or ED visits for HF, and an increase in LVEDD and all‐cause death, unadjusted and adjusted for age and sex

**Figure S4:** The association between an increase in LVEDD and HF hospitalization, and LVEDD and ED visits for HF, unadjusted and age and sex‐adjusted models

**Figure S5:** The association between the absolute change in LVESD and the composite of all‐cause death, HF hospitalization, or ED visits for HF

**Figure S6:** The association between the absolute change in LVEDD and the composite of all‐cause death, HF hospitalization, or ED visits for HF

## Data Availability

To comply with Alberta's Health Information Protection Act, the data set for this study is not publicly available.
