# Supplementary material for: Prognostic Significance and Factors Associated With Worsening LV Dilatation in Heart Failure
Source: Clin Cardiol. 2026 Oct 7;49(10):e70490. doi: 10.1002/clc.70490 (PMC13644549; doi:10.1002/clc.70490)
Supplement: Supplementary file 1 [file CLC-49-e70490-s002.docx]

**Supplementary material**

**Supplementary table S1: Association between an increase in LVESD and clinical outcomes**

| **Variable** | **All-cause death**  **Hazard ratio**  **(95% CI)** | **HF hospitalization**  **Hazard ratio**  **(95% CI)** | **ED visits for HF**  **Hazard ratio**  **(95% CI)** |
| --- | --- | --- | --- |
| LVESD increase | 1.04 (0.98, 1.12) | 1.27 (1.15, 1.39) | 1.25 (1.15, 1.37) |
| Index LVEF | 1.00 (0.99, 1.00) | 1.01 (1.00, 1.01) | 1.01 (1.00, 1.01) |
| Age | 1.04 (1.03, 1.04) | 1.17 (1.10, 1.24) | 1.18 (1.11, 1.25) |
| Male | 1.03 (0.96, 1.10) | 1.03 (1.02, 1.03) | 1.02 (1.02, 1.03) |
| Index LVESD | 1.00 (0.95, 1.04) | 0.98 (0.89, 1.08) | 0.95 (0.87, 1.04) |
| Ischemic heart disease | 1.13 (1.06, 1.22) | 1.13 (1.03, 1.24) | 1.05 (0.96, 1.15) |
| Atrial fibrillation | 1.23 (1.15, 1.31) | 1.11 (1.01, 1.22) | 1.09 (1.00, 1.19) |
| Index mitral regurgitation severity |  |  |  |
| Mild, mild to moderate | 1.07 (1.00, 1.15) | 1.10 (1.00, 1.21) | 1.07 (0.97, 1.17) |
| Moderate, moderate to severe | 1.22 (1.10, 1.36) | 1.20 (1.03, 1.39) | 1.14 (0.99, 1.31) |
| Severe | 1.08 (0.84, 1.39) | 0.90 (0.64, 1.26) | 0.95 (0.69, 1.30) |
| Chronic obstructive pulmonary disease | 1.49 (1.39, 1.60) | 1.10 (0.99, 1.21) | 1.14 (1.04, 1.25) |
| Chronic kidney disease | 1.78 (1.67, 1.91) | 1.09 (0.99, 1.19) | 1.13 (1.02, 1.25) |
| Hypertension | 0.97 (0.89, 1.05) | 1.14 (1.02, 1.28) | 1.04 (0.95, 1.14) |
| Dementia | 1.58 (1.41, 1.76) | 0.70 (0.57, 0.86) | 0.62 (0.51, 0.76) |
| Cancer (any) | 1.51 (1.37, 1.67) | 0.65 (0.54, 0.78) | 0.72 (0.61, 0.84) |
| Anemia | 1.21 (1.10, 1.33) | 0.98 (0.85, 1.14) | 1.00 (0.88, 1.15) |
| Diabetes | 1.31 (1.22, 1.40) | 1.31 (1.19, 1.44) | 1.32 (1.21, 1.45) |
| Depression | 1.15 (1.04, 1.28) | 1.15 (0.98, 1.34) | 1.02 (0.88, 1.19) |
| CRT | 0.63 (0.33, 1.22) | 1.39 (0.74, 2.63) | 1.66 (0.98, 2.80) |
| ICD | 0.81 (0.65, 1.00) | 0.98 (0.77, 1.26) | 0.92 (0.72, 1.17) |
| Permanent pacemaker | 0.90 (0.77, 1.04) | 1.07 (0.88, 1.31) | 1.17 (0.98, 1.39) |
| Prescribed any ACE inhibitor or ARB at follow-up | 0.81 (0.75, 0.87) | 1.25 (1.11, 1.40) | 1.19 (1.07, 1.32) |
| Prescribed any beta-blocker at follow-up | 0.84 (0.78, 0.91) | 0.98 (0.88, 1.10) | 0.99 (0.90, 1.10) |

ACE: Angiotensin converting enzyme; ARB: angiotensin receptor blocker; CRT: cardiac resynchronization therapy; ICD: implantable cardioverter-defibrillator; LVEF: left ventricular ejection fraction; LVESD: Left ventricular end systolic dimension

An increase in LVESD was defined as an increase ≥-3.13% between echocardiograms ≥6 months apart

**Supplementary table S2: Association between LVEDD and clinical outcomes**

| **Variable** | **All-cause death**  **Hazard ratio**  **(95% CI)** | **HF hospitalization**  **Hazard ratio**  **(95% CI)** | **ED visits for HF**  **Hazard ratio**  **(95% CI)** |
| --- | --- | --- | --- |
| LVEDD | 1.01 (0.94, 1.08) | 1.24 (1.12, 1.36) | 1.16 (1.06, 1.26) |
| Index LVEF | 0.99 (0.99, 1.00) | 1.00 (1.00, 1.01) | 1.00 (1.00, 1.01) |
| Age | 1.04 (1.03, 1.04) | 1.16 (1.08, 1.24) | 1.19 (1.12, 1.27) |
| Male | 1.05 (0.98, 1.13) | 1.03 (1.02, 1.03) | 1.02 (1.02, 1.03) |
| Index LVEDD | 0.93 (0.89, 0.98) | 0.98 (0.89, 1.08) | 0.94 (0.86, 1.04) |
| Ischemic heart disease | 1.13 (1.06, 1.22) | 1.13 (1.03, 1.25) | 1.06 (0.97, 1.16) |
| Atrial fibrillation | 1.22 (1.14, 1.31) | 1.11 (1.01, 1.22) | 1.09 (1.00, 1.19) |
| Index mitral regurgitation severity |  |  |  |
| Mild, mild to moderate | 1.08 (1.01, 1.16) | 1.11 (1.00, 1.22) | 1.06 (0.97, 1.17) |
| Moderate, moderate to severe | 1.24 (1.12, 1.38) | 1.21 (1.04, 1.40) | 1.14 (0.99, 1.31) |
| Severe | 1.14 (0.88, 1.46) | 0.92 (0.65, 1.29) | 0.95 (0.69, 1.30) |
| Chronic obstructive pulmonary disease | 1.50 (1.40, 1.60) | 1.09 (0.99, 1.21) | 1.13 (1.03, 1.24) |
| Hypertension | 0.97 (0.90, 1.05) | 1.14 (1.02, 1.27) | 1.13 (1.01, 1.25) |
| Chronic kidney disease | 1.78 (1.67, 1.91) | 1.09 (0.99, 1.19) | 1.04 (0.95, 1.14) |
| Dementia | 1.56 (1.40, 1.75) | 0.70 (0.57, 0.86) | 0.62 (0.51, 0.76) |
| Cancer | 1.50 (1.36, 1.66) | 0.64 (0.54, 0.77) | 0.71 (0.60, 0.83) |
| Anemia | 1.22 (1.11, 1.34) | 0.98 (0.85, 1.13) | 1.00 (0.88, 1.15) |
| Diabetes | 1.31 (1.23, 1.41) | 1.31 (1.19, 1.44) | 1.32 (1.21, 1.44) |
| Depression | 1.15 (1.03, 1.27) | 1.14 (0.98, 1.33) | 1.02 (0.87, 1.18) |
| CRT | 0.66 (0.34, 1.27) | 1.42 (0.75, 2.70) | 1.67 (0.99, 2.80) |
| ICD | 0.83 (0.67, 1.02) | 1.00 (0.78, 1.29) | 0.94 (0.74, 1.20) |
| Permanent pacemaker | 0.90 (0.78, 1.05) | 1.07 (0.88, 1.30) | 1.17 (0.98, 1.39) |
| Prescribed any ACE inhibitor or ARB at follow-up | 0.81 (0.76, 0.88) | 1.24 (1.10, 1.38) | 1.18 (1.06, 1.31) |
| Prescribed any beta-blocker at follow-up | 0.85 (0.79, 0.91) | 0.99 (0.88, 1.10) | 0.99 (0.90, 1.10) |

ACE: Angiotensin converting enzyme; ARB: angiotensin receptor blocker; CRT: cardiac resynchronization therapy; ICD: implantable cardioverter-defibrillator; LVEDD: Left ventricular end diastolic dimension; LVEF: left ventricular ejection fraction; MRA: mineralocorticoid receptor antagonist

An increase in LVEDD was defined as a ≥1.33% increase in LVEDD between echocardiograms ≥6 months apart.

**Supplementary table S3: The association between a 3mm increase in LVESD and clinical outcomes**

| **Variable** | **Composite outcome**  **Hazard ratio (95% CI)** | **All-cause death Hazard ratio (95% CI)** | **HF hospitalization, Hazard ratio (95% CI)** | **ED visits for HF, Hazard ratio (95% CI)** |
| --- | --- | --- | --- | --- |
| 3mm increase in LVESD | 1.09 (1.02, 1.15) | 0.99 (0.93, 1.06) | 1.22 (1.11, 1.34) | 1.15 (1.05, 1.26) |
| Index LVEF | 1.00 (1.00, 1.00) | 1.00 (0.99, 1.00) | 1.01 (1.00, 1.01) | 1.00 (1.00, 1.01) |
| Index LVESD | 1.05 (1.01, 1.09) | 0.99 (0.94, 1.03) | 1.16 (1.09, 1.23) | 1.16 (1.10, 1.23) |
| Age | 1.03 (1.03, 1.03) | 1.03 (1.03, 1.04) | 1.03 (1.02, 1.03) | 1.02 (1.02, 1.03) |
| Male sex | 0.98 (0.92, 1.04) | 1.03 (0.96, 1.10) | 0.98 (0.89, 1.08) | 0.96 (0.88, 1.05) |
| Ischemic heart disease | 1.12 (1.05, 1.19) | 1.14 (1.06, 1.22) | 1.13 (1.02, 1.24) | 1.05 (0.96, 1.15) |
| Atrial fibrillation | 1.23 (1.16, 1.31) | 1.23 (1.15, 1.31) | 1.11 (1.01, 22) | 1.08 (0.99, 1.18) |
| Index mitral regurgitation severity |  |  |  |  |
| Mild, mild-moderate | 1.08 (1.02, 1.15) | 1.07 (1.00, 1.15) | 1.10 (1.00, 1.21) | 1.06 (0.97, 1.17) |
| Moderate, moderate to severe | 1.24 (1.13, 1.36) | 1.22 (1.10, 1.36) | 1.19 (1.03, 1.38) | 1.14 (1.00, 1.31) |
| Severe | 1.04 (0.84, 1.29) | 1.08 (0.84, 1.39) | 0.90 (0.64, 1.27) | 0.96 (0.70, 1.31) |
| Chronic obstructive pulmonary disease | 1.40 (1.32, 1.49) | 1.49 (1.40, 1.60) | 1.10 (0.99, 1.21) | 1.13 (1.03, 1.24) |
| Hypertension | 1.02 (0.95, 1.10) | 0.97 (0.90, 1.05) | 1.14 (1.02, 1.28) | 1.13 (1.01, 1.25) |
| Chronic kidney disease | 1.57 (1.48, 1.67) | 1.78 (1.67, 1.91) | 1.09 (0.99, 1.20) | 1.04 (0.96, 1.14) |
| Dementia | 1.32 (1.19, 1.47) | 1.57 (1.40, 1.76) | 0.70 (0.57, 0.85) | 0.62 (0.51, 0.76) |
| Cancer (any) | 1.31 (1.20, 1.44) | 1.51 (1.37, 1.66) | 0.65 (0.54, 0.77) | 0.71 (0.61, 0.84) |
| Anemia | 1.19 (1.09, 1.30) | 1.21 (1.11, 1.33) | 0.98 (0.85, 1.14) | 1.00 (0.88, 1.15) |
| Diabetes | 1.37 (1.29, 1.45) | 1.31 (1.22, 1.40) | 1.31 (1.19, 1.44) | 1.32 (1.21, 1.44) |
| Depression | 1.15 (1.05, 1.26) | 1.15 (1.03, 1.28) | 1.14 (0.97, 1.32) | 1.01 (0.87, 1.18) |
| CRT | 1.03 (0.64, 1.64) | 0.64 (0.33, 1.24) | 1.40 (0.74, 1.28) | 1.68 (0.99, 2.83) |
| ICD | 0.82 (0.69 0.98) | 0.82 (0.66, 1.01) | 1.00 (0.78, 1.28) | 0.94 (0.84, 1.20) |
| Pacemaker | 0.95 (0.84, 1.08) | 0.90 (0.77, 1.04) | 1.08 (9.88, 1.31) | 1.18 (0.99, 1.40) |
| Prescribed any ACE inhibitor or ARB at follow-up | 0.90 (0.85, 0.96) | 0.81 (0.75, 0.87) | 1.24 (1.11, 1.39) | 1.19 (1.07, 1.32) |
| Prescribed any beta blocker at follow-up | 0.87 (0.82, 0.93) | 0.84 (0.78, 0.91) | 0.98 (0.88, 1.10) | 0.99 (0.90, 1.10) |

**Supplementary table S4: The association between a 3mm increase in LVEDD and clinical outcomes**

| **Variable** | **Composite outcome**  **Hazard ratio (95% CI)** | **All-cause death Hazard ratio (95% CI)** | **HF hospitalization, Hazard ratio (95% CI)** | **ED visits for HF, Hazard ratio (95% CI)** |
| --- | --- | --- | --- | --- |
| 3mm increase in LVEDD | 1.06 (1.00, 1.13) | 0.97 (0.90, 1.04) | 1.21 (1.10, 1.34) | 1.19 (1.08, 1.30) |
| Index LVEF | 1.00 (1.00, 1.00) | 0.99 (0.99, 1.00) | 1.00 (1.00, 1.00) | 1.00 (1.00, 1.01) |
| Index LVEDD | 1.01 (0.97, 1.05) | 0.93 (0.88, 0.97) | 1.15 (1.07, 1.23) | 1.19 (1.12, 1.27) |
| Age | 1.03 (1.03, 1.03) | 1.03 (1.03, 1.04) | 1.03 (1.02, 1.03) | 1.02 (1.02, 1.03) |
| Male sex | 1.00 (0.94, 1.06) | 1.05 (0.98, 1.13) | 0.98 (0.89, 1.08) | 0.94 (0.86, 1.03) |
| Ischemic heart disease | 1.12 (1.06, 1.19) | 1.14 (1.06, 1.22) | 1.14 (1.03, 1.25) | 1.06 (0.97, 1.16) |
| Atrial fibrillation | 1.23 (1.16, 1.30) | 1.22 (1.14, 1.31) | 1.11 (1.01, 1.22) | 1.09 (1.00, 1.19) |
| Index mitral regurgitation severity |  |  |  |  |
| Mild, mild-moderate | 1.09, (1.02, 1.16) | 1.08 (1.01, 1.16) | 1.11 (1.00, 1.22) | 1.06 (0.97, 1.17) |
| Moderate, moderate to severe | 1.26 (1.14, 1.38) | 1.24 (1.12, 1.38) | 1.21 (1.05, 1.40) | 1.15 (1.00, 1.32) |
| Severe | 1.07 (0.86, 1.33) | 1.14 (0.89, 1.46) | 0.92 (0.65, 1.29) | 0.94 (0.68, 1.29) |
| Chronic obstructive pulmonary disease | 1.40 (1.32, 1.49) | 1.50 (1.40, 1.60) | 1.09 (0.99, 1.20) | 1.13 (1.03, 1.24) |
| Hypertension | 1.02 (0.95, 1.10) | 0.97 (0.90, 1.06) | 1.14 (1.01, 1.27) | 1.12 (1.01, 1.25) |
| Chronic kidney disease | 1.57 (1.48, 1.67) | 1.78 (1.67, 1.91) | 1.09 (0.99, 1.19) | 1.04 (0.95, 1.13) |
| Dementia | 1.32 (1.19, 1.46) | 1.56 (1.39, 1.74) | 0.70 (0.57, 0.85) | 0.62 (0.51, 0.76) |
| Cancer (any) | 1.31 (1.20, 1.43) | 1.50 (1.36, 1.65) | 0.65 (0.54, 0.77) | 0.71 (0.61, 0.84) |
| Anemia | 1.19 (1.10, 1.30) | 1.22 (1.11, 1.34) | 0.98 (0.85, 1.13) | 1.00 (0.87, 1.14) |
| Diabetes | 1.37 (1.29, 1.45) | 1.31 (1.23, 1.41) | 1.31 (1.19, 1.44) | 1.32 (1.21, 1.44) |
| Depression | 1.15 (1.04, 1.26) | 1.15 (1.03, 1.28) | 1.13 (0.97, 1.32) | 1.01 (0.87, 1.17) |
| CRT | 1.05 (0.66, 1.67) | 0.67 (0.35, 1.29) | 1.40 (0.84, 2.66) | 1.64 (0.98, 2.75) |
| ICD | 0.84 (0.70, 1.00) | 0.83 (0.68, 1.03) | 1.00 (0.78, 1.29) | 0.93 (0.73, 1.19) |
| Pacemaker | 0.95 (0.84, 1.09) | 0.91 (0.78, 1.05) | 1.07 (0.88, 1.31) | 1.17 (0.98, 1.40) |
| Prescribed any ACE inhibitor or ARB at follow-up | 0.80 (0.85, 0.97 | 0.82 (0.76, 0.88) | 1.24 (1.10, 1.38) | 1.18 (1.07, 1.31) |
| Prescribed any beta blocker at follow-up | 0.87 (0.82, 0.93) | 0.85 (0.78, 0.91) | 0.99 (0.89, 1.10) | 1.00 (0.90, 1.10) |

**Supplementary figure title and legends**

**Supplementary figure S1: The association between an increase in LVESD and composite all-cause death, HF readmission, or ED visits for HF, and an increase in LVESD and death, unadjusted and adjusted for age and sex**

ED: Emergency department; HF: heart failure; LVESD: Left ventricular end-systolic dimension

A) Unadjusted Kaplan-Meier curve for the composite all-cause death, HF readmission, or ED visits for HF separated by an increase in LVESD; B) Kaplan-Meier curve for the composite outcome, separated by an increase in LVESD, adjusted for age and sex; C) Unadjusted Kaplan-Meier curve for all-cause death separated by an increase in LVESD; D) Kaplan-Meier curve for all-cause death, separated by an increase in LVESD, adjusted for age and sex

An increase in LVESD was defined as an increase ≥-3.13% between echocardiograms ≥6 months apart

**Supplementary figure S2: The association between an increase in LVESD and HF hospitalization, and an increase in LVESD and ED visits for HF, unadjusted and age and sex-adjusted models**

ED: Emergency department; HF: heart failure; LVESD: Left ventricular end-systolic dimension

A) Unadjusted Kaplan-Meier curve for HF hospitalization separated by an increase in LVESD; B) Kaplan-Meier curve for HF hospitalization, separated by an increase in LVESD, adjusted for age and sex; C) Unadjusted Kaplan-Meier curve for ED visits for HF separated by an increase in LVESD; D) Kaplan-Meier curve for ED visits for HF, separated by an increase in LVESD, adjusted for age and sex

An increase in LVESD was defined as an increase ≥-3.13% between echocardiograms ≥6 months apart

**Supplementary figure S3: The association between an increase in LVEDD and composite all-cause death, HF readmission, or ED visits for HF, and an increase in LVEDD and all-cause death, unadjusted and adjusted for age and sex**

ED: Emergency department; HF: heart failure; LVEDD: Left ventricular end diastolic dimension

A) Unadjusted Kaplan-Meier curve for the composite all-cause death, HF readmission, or ED visits for HF separated by an increase in LVEDD; B) Kaplan-Meier curve for the composite outcome, separated by an increase in LVEDD, adjusted for age and sex; C) Unadjusted Kaplan-Meier curve for all-cause death separated by an increase in LVEDD; D) Kaplan-Meier curve for all-cause death, separated by an increase in LVEDD, adjusted for age and sex

An increase in LVEDD was defined as a ≥1.33% increase in LVEDD between echocardiograms ≥6 months apart.

**Supplementary figure S4: The association between an increase in LVEDD and HF hospitalization, and LVEDD and ED visits for HF, unadjusted and age and sex-adjusted models**

ED: Emergency department; HF: heart failure; LVEDD: Left ventricular end diastolic dimension

A) Unadjusted Kaplan-Meier curve for HF hospitalization separated by an increase in LVEDD; B) Kaplan-Meier curve for HF hospitalization, separated by an increase in LVEDD, adjusted for age and sex; C) Unadjusted Kaplan-Meier curve for ED visits for HF separated by an increase in LVEDD; D) Kaplan-Meier curve for ED visits for HF, separated by an increase in LVEDD, adjusted for age and sex

An increase in LVEDD was defined as a ≥1.33% increase in LVEDD between echocardiograms ≥6 months apart.

**Supplementary figure S5: The association between the absolute change in LVESD and the composite of all-cause death, HF hospitalization, or ED visits for HF**

ED: Emergency department; HF: heart failure; LVESD: left ventricular end systolic dimension

The analysis was performed using restricted cubic splines in a fully adjusted model

**Supplementary figure S6: The association between the absolute change in LVEDD and the composite of all-cause death, HF hospitalization, or ED visits for HF**

ED: Emergency department; HF: heart failure; LVEDD: left ventricular end diastolic dimension

The analysis was performed using restricted cubic splines in fully adjusted models.
